# Hepatoma SK Hep-1 Cells Exhibit Characteristics of Oncogenic Mesenchymal Stem Cells with Highly Metastatic Capacity

**DOI:** 10.1371/journal.pone.0110744

**Published:** 2014-10-22

**Authors:** Jong Ryeol Eun, Yong Jin Jung, Yanling Zhang, Yanhong Zhang, Benjamin Tschudy-Seney, Rajen Ramsamooj, Yu-Jui Yvonne Wan, Neil D. Theise, Mark A. Zern, Yuyou Duan

**Affiliations:** 1 Department of Internal Medicine, University of California Davis Medical Center, Sacramento, California, United States of America; 2 Institute for Regenerative Cures, University of California Davis Medical Center, Sacramento, California, United States of America; 3 Department of Pathology and Laboratory Medicine, University of California Davis Medical Center, Sacramento, California, United States of America; 4 Department of Internal Medicine, Yeungnam University College Medicine, Daegu, Korea; 5 Department of Internal Medicine, SMG-SNU Boramae Medical Center, Seoul National University College of Medicine, Seoul, Korea; 6 School of Biotechnology, Southern Medical University, Guangzhou, China; 7 Department of Pathology and Medicine, Beth Israel Medical Center, Albert Einstein College of Medicine, New York, New York, United States of America; The University of Tennessee Health Science Center, United States of America

## Abstract

**Background:**

SK Hep-1 cells (SK cells) derived from a patient with liver adenocarcinoma have been considered a human hepatoma cell line with mesenchymal origin characteristics, however, SK cells do not express liver genes and exhibit liver function, thus, we hypothesized whether mesenchymal cells might contribute to human liver primary cancers. Here, we characterized SK cells and its tumourigenicity.

**Methods and Principal Findings:**

We found that classical mesenchymal stem cell (MSC) markers were presented on SK cells, but endothelial marker CD31, hematopoietic markers CD34 and CD45 were negative. SK cells are capable of differentiate into adipocytes and osteoblasts as adipose-derived MSC (Ad-MSC) and bone marrow-derived MSC (BM-MSC) do. Importantly, a single SK cell exhibited a substantial tumourigenicity and metastatic capacity in immunodefficient mice. Metastasis not only occurred in circulating organs such as lung, liver, and kidneys, but also in muscle, outer abdomen, and skin. SK cells presented greater in vitro invasive capacity than those of Ad-MSC and BM-MSC. The xenograft cells from subcutaneous and metastatic tumors exhibited a similar tumourigenicity and metastatic capacity, and showed the same relatively homogenous population with MSC characteristics when compared to parental SK cells. SK cells could unlimitedly expand in vitro without losing MSC characteristics, its tumuorigenicity and metastatic capacity, indicating that SK cells are oncogenic MSC with enhanced self-renewal capacity. We believe that this is the first report that human MSC appear to be transformed into cancer stem cells (CSC), and that their derivatives also function as CSCs.

**Conclusion:**

Our findings demonstrate that SK cells represent a transformation mechanism of normal MSC into an enhanced self-renewal CSC with metastasis capacity, SK cells and their xenografts represent a same relative homogeneity of CSC with substantial metastatic capacity. Thus, it represents a novel mechanism of tumor initiation, development and metastasis by CSCs of non-epithelial and endothelia origin.

## Introduction

Carcinomas are a heterogeneous population of a series of cells with different phenotypes; however, the origin of many cancers remains unknown. Human liver carcinoma is the fifth most common cancer worldwide [1], and there are two major nonexclusive hypotheses of the cellular origin of the liver cancers: that they derive from stem cells due to maturation arrest or from dedifferentiation of mature cells. Phenotypically liver cancers can be divided into well-, moderate-, and poorly-differentiated carcinomas, and liver-specific genes can be detected in well- and moderately-differentiated liver cancer cells, indicating that these cancers originated from hepatocytes and cholangiocytes; however, liver specific genes cannot be detected in poorly-differentiated liver cancer cells, suggesting that these cancers might originate from other cell types rather than hepatocytes and cholangiocytes. A third possibility has also been suggested for rodent and human hepatic malignancies: that bone marrow derived cells may incorporate into benign or malignant liver tumors [2] or actually initiate tumor development [3]. In adult liver, 78% of the cells are hepatocytes; and the majority of non-hepatocytes are thought to be cholangiocytes [4], sinusoidal endothelia cells, Kupffer cells, hepatobiliary stem/progenitor cells, hepatic stellate cells, myofibroblasts, and pit cells [5]–[9]. They are implicated in the physiological and pathophysiological process of liver regeneration, injury and repair. In humans, besides hepatocytes and cholangiocytes, only endothelia cells has been reported to be the origin of human liver carcinomas [10]; i.e. the SK Hep-1 (SK) cell line. SK cell is a human cell line derived from a patient with liver adenocarcinoma in 1971 [11], and it has been reported to be of endothelia origin with mesenchymal characteristics in 1992 [10]. In our study, we found that the morphology, the tubular formation, and the expression of vimentin (mesenchymal origin) in SK cells are the same as in previous reports [10], [12]; however, our investigation further revealed that SK cells did not CD31, a typical endothelial marker, importantly, endothelia cells originate from mesenchymal origin [13]. Thus, we hypothesize whether SK cells represent a kind of mesenchymal cell type, and demonstrate whether mesenchymal cells contribute to human primary liver cancer. In this study, we characterized SK cells, and found that SK cells exhibited an oncogenic mesenchaymal stem cell line with a great metastatic capacity.

## Materials and Methods

### Cell lines and cell culture

SK Hep-1 cell, a hepatoma cell line, was purchased from ATCC (www.atcc.org); adipose-derived mesenchymal stem cells (MSC-ad) and bone marrow-derived mesenchymal stem cells (MSC-BM) were purchased from ScienCell Research Laboratories (www.sciencellonline.com). The cell culture conditions for growing and expanding these line cells were per the manufacturers instructions.

### Flow cytometry analysis for surface markers

The immunophenotypic characterizations of SK cells, MSC-ad, MSC-BM, and the re-cultured cells from the isolation of tumor xenografts and metastatic tumors were performed by flow cytometry employing 22 PE conjugated antibodies: CD90 (Thy-1 cell surface antigen), CD44, CD73 (NT5E, 5′-nucleotidase), CD71 (Transferrin receptor protein 1 (TfR1)), CD105 (endoglin, ENG), CD166 (activated leukocyte cell adhesion molecule, ALCAM), CD29 (integrin, beta-1, ITGB1), CD106 (vascular cell adhesion molecule 1, VCAM1), CD49d (integrin alpha subunit), CD146 (melanoma cell adhesion molecule, MCAM), CD133 (Prominin 1, PROM1), CD13 (Aminopeptidase N), CD54 ICAM-1 (Intercellular Adhesion Molecule 1, ICAM-1), CD9, CD10 (membrane metallo-endopeptidase), ABC (major histocompatibility complex (MHC) class I antigen), HLA DR (major histocompatibility complex (MHC) class II antigen), and Alkaline phosphatase (ALP), CD31 (endothelia cell marker), CD34 and CD45 (hematopoietic markers). The cells were stained as previously described [14]; all antibodies used are listed in Table S1.

### Differentiation capacity of SK cells

We evaluated the potential of SK cells, MSC-ad, and MSC-BM to differentiate into adipocytes and osteocytes. The differentiation and staining with red oil R and alizarin red S was performed as previously described [15]. ALP activity was assessed by NBT/BICP staining (Roche).

### The generation of cDNA

RNA was extracted from the SK cells, xenograft cells, as well as undifferentiated and differentiated MSC cells using the Qiagen mini RNA kit, and cDNA was generated as previously described [14]. All primers/probes used are listed in the Table S2.

### Tumorigenicity of SK cells

SK cells were injected into 8–10 weeks, female NOD/SCID/IL2rg mice (Jackson Laboratory) by subcutaneous injection of 1, 10, 100, 500, 1,000, 5,000, or 10,000 cells. The re-cultured cells from primary subcutaneous tumor and metastatic tumors produced by the injection of parental SK cells were also injected into the same mouse models with 10 and 100 cells. The small number of cells (1 and 10) was mixed with 30% of Matrigel (BD Sciences) for the purpose of avoiding to lose cells during preparation and transplantation [16]. Surgical procedures for transplantation and monitoring the tumor formation and subsequent tumor collection were approved by the Animal Care and Use Administrative Advisory Committee of the University of California Davis.

### The isolation and re-culture of the tumor xenograft cells

The tumors xenografts were harvested and cut into small piece under sterile conditions, and treated with dispase (1 mg/ml) and collagenase type IV (1 mg/ml), and incubated at 37C for 20 min, then the tumor tissue was homogenized with a serological pipette, and supernatants were collected. The remaining tissue was treated with the same solution for an additional 2–4 times until almost all tissue was digested. The supernatants were spun at 300 g for 5 min, the cell pellet was re-suspended with MEM medium after discarding the supernatant and was treated with fixative-free lysing solution (www.invitrogen.com) for 10 min in the dark to destroy the blood cells, then filtered with a 100 um cell strainer and spun again. Finally the cell pellet were re-suspended, and re-cultured with MEM (parental SK cell culture condition).

### Histological assay of xenografts

Primary xenografts and organ tissue with metastatic tumor xenografts were collected and fixed with 10% formalin, and used for hematoxylin and eosin staining (H&E stain).

### In vitro invasion assay

In vitro migration and invasion was performed using a basement membrane-based invasion assay (Cell Biolabs, Inc). 100,000 SK cells or MSC-ad or MSC-BM were cultured in a 24-well size transwell containing growth medium with low serum (2% of FBS), while the bottom wells were filled with normal growth medium containing 10% of FBS. After 24 hours culturing, non-invasive cells were removed, and invasive cells that passed through the basement membrane were stained and imaged. In a parallel experiment, the invasive cells were stained and quantified by measuring OD 560 nm values. 20000, 40000, 60000, 80000, and 100000 SK cells and MSC were seeded in the bottom well, and stained and quantified as a standard curve. The procedures of the invasion assay were performed per the manufacturer's instructions.

### Statistics

All data were summarized as means ± SEM from at least three independent measurements. An unpaired Student t test was used to analyze the data. p<0.05 was considered statistically significant.

## Results

### SK cells exhibited an MSC phenotype

**Figure 1:**
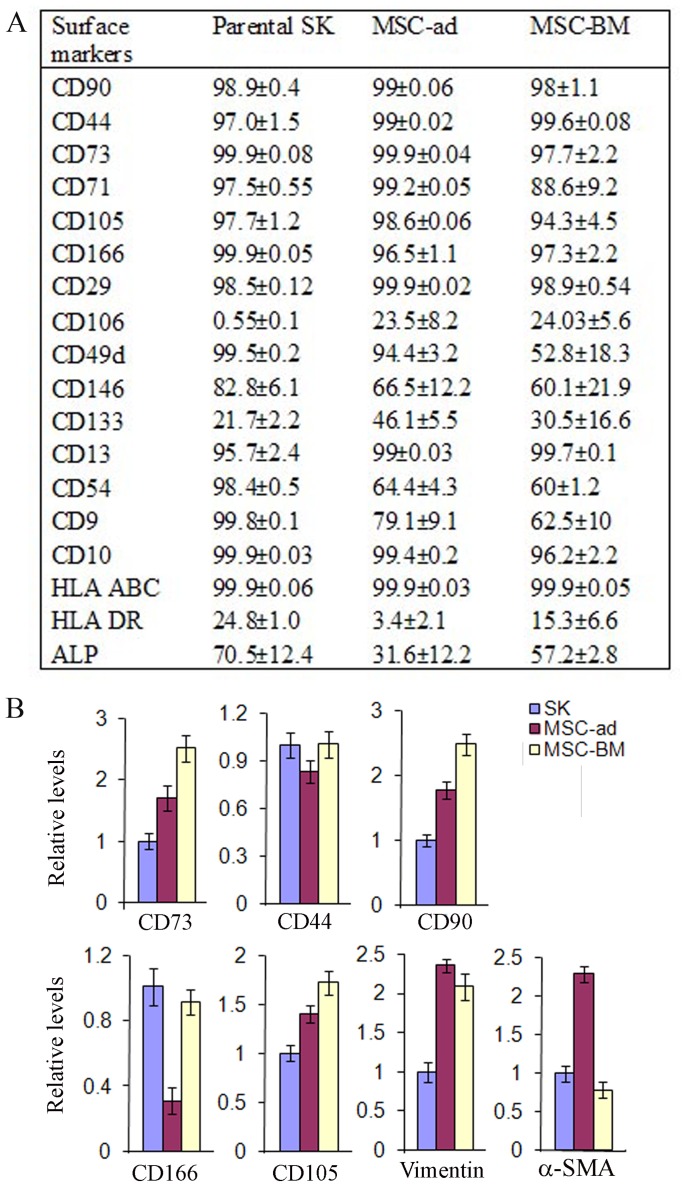
Characterization and comparison of SK cells with mesenchymal stem cells. A, flow cytometry was employed to detect classical mesenchymal stem cell (MSC) markers in three cell types, SK Hep-1 cells (SK cells), adipose-derived MSC (MSC-ad), and bone marrow-derived MSC (MSC-BM). B, expression of MSC markers, vimentin (a mesodermal origin marker), and alpha smooth muscle actin (α-SMA) was measured by quantitative PCR in three cell types. Abbreviations: HLA ABC, major histocompatibility complex (MHC) class I antigen; HLA DR, MHC class II antigen; ALP, Alkaline phosphatase.

We compared surface markers of SK cells to mesenchymal stem cells isolated from adipose and bone marrow (MSC-ad, MSC-BM). We found that classical MSC markers, such as CD90, CD44, CD73, CD71, CD105, CD146, CD166, CD29, CD13, CD9, CD10, HLA ABC, and ALP were also present on SK cells (Figure 1A, Figure S1); however, CD106 was almost absent in SK cells. Hematopoietic markers, CD34, and CD45, and endothelia marker CD31, were negative in SK cells, MSC-ad and MSC-BM (data not shown). Expression levels of MSC markers CD73, CD44, CD90, CD105, CD166, α-SMA, and the mesenchymal origin marker, vimentin, did not exhibit significant biological difference among the three cell type (Figure 1B).

### Adipogenic and osteogenic differentiation of SK cells

**Figure 2:**
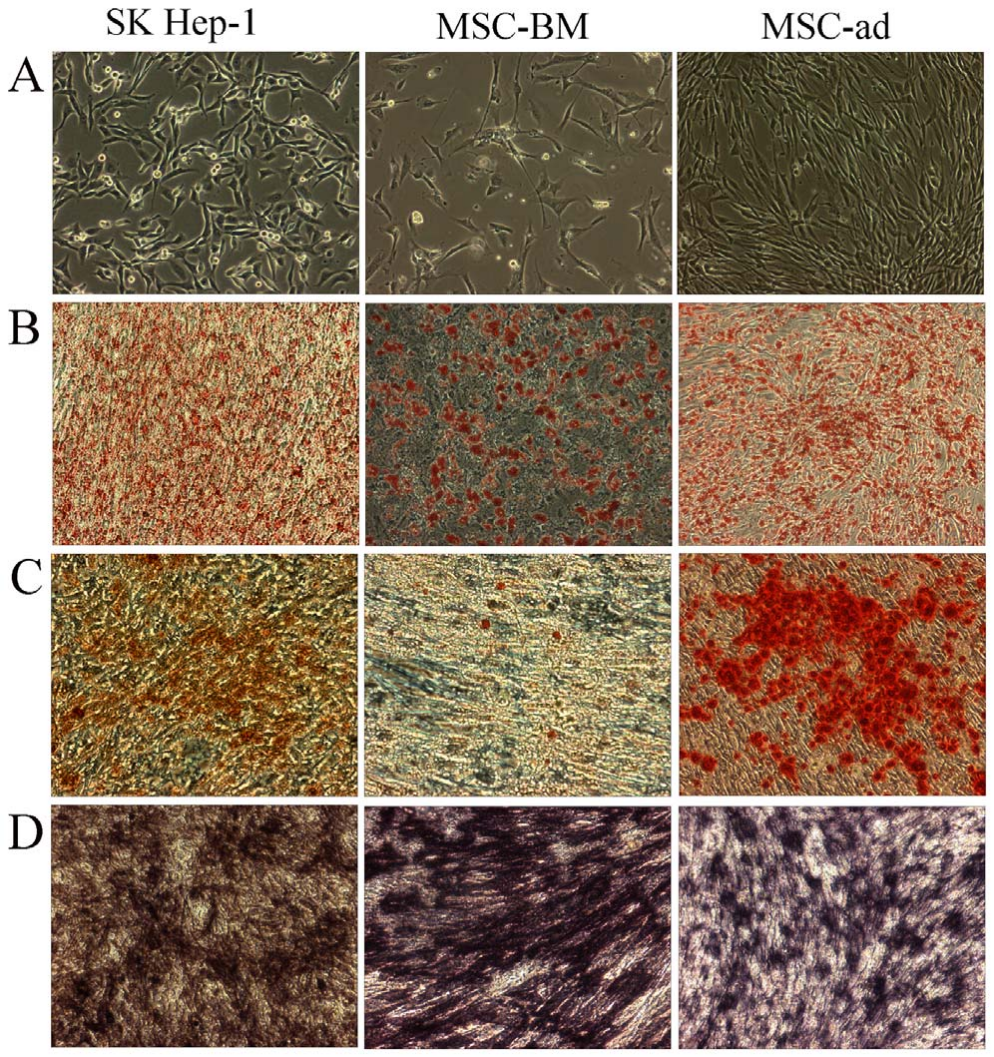
Differentiation of three cell types into adipocytes and osteoblasts. A, SK cells, adipocyte-derived MSC (MSC-ad), and bone marrow-derived MSC (MSC-BM) were used for the differentiation. B, the three cell types were differentiated into adipocytes determined by Oil Red R staining. C, the three cell types were differentiated into osteoblasts determined by Alizarin Red S staining. D, alkaline phosphatase staining was performed after the osteoblast differentiation. Magnifications: 100X

**Figure 3:**
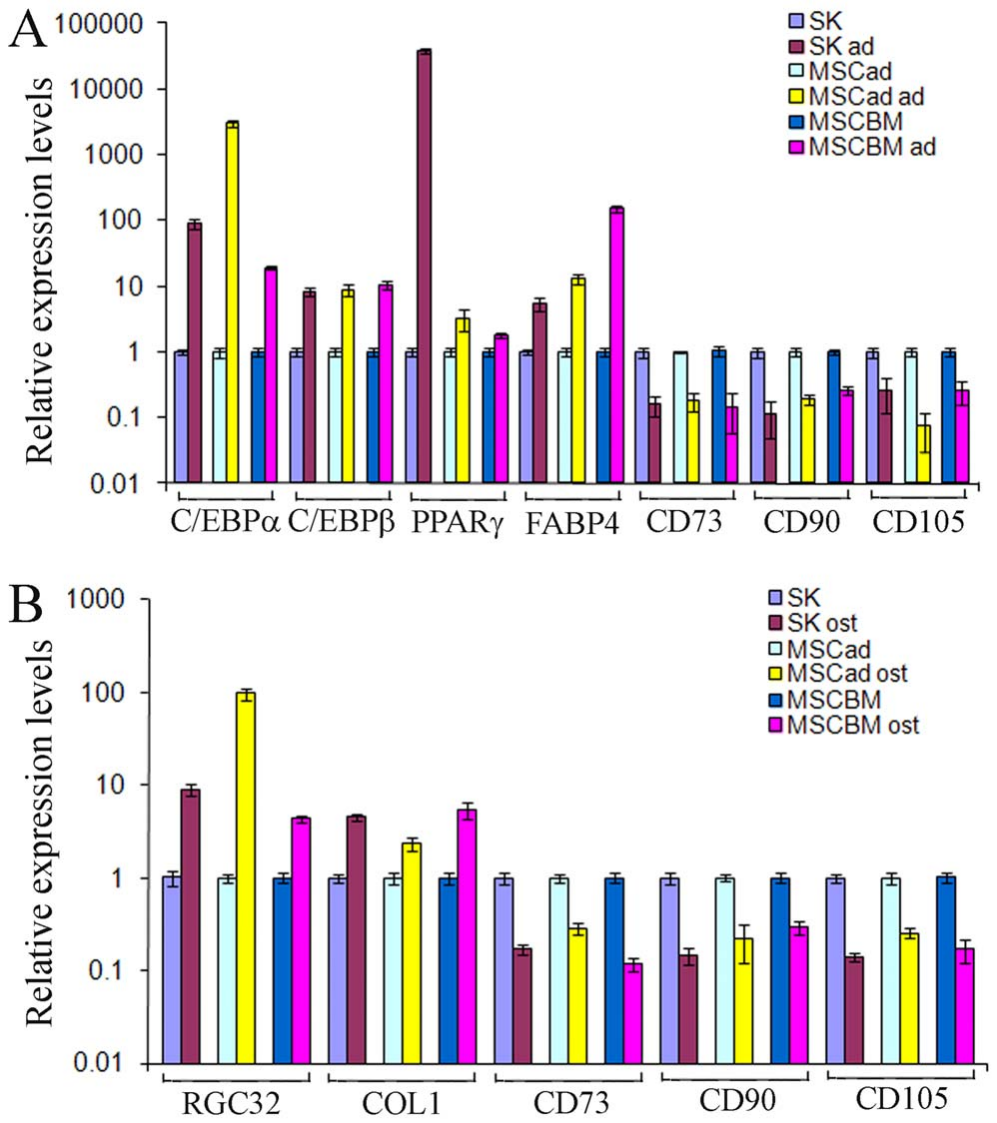
Gene expression patterns in differentiated SK cells, MSC-ad and MSC-BM. A and B, expression levels of adipocyte genes, C/EBPα, C/EBPβ, PPAR-γ, and FABP4 (A), osteoblast genes, RBC32 and COL1 (B), and MSC genes, CD73, CD90, and CD105 (A and B) were measured by quantitative PCR in differentiated SK cells, MSC-ad and MSC-BM at day 15 after differentiation. Abbreviations: SK, SK Hep-1 cells; MSC-ad, adipocyte-derived MSC; MSC-BM, bone marrow-derived MSC; FABP4, fatty acid binding protein 4; RBC32, response gene to complement 32; COL1, collage 1A1.

After culturing with adipogenic or osteogenic differentiation medium for 2 weeks, SK cells were differentiated into adipocytes and osteoblasts, with a similar differentiation pattern as MSC-ad and MSC-BM do, thereby confirming its MSC-like characteristics. Adipogenic differentiation was evidenced by Red oil R staining, whereas Alizarin red S staining confirmed the accumulation of calcium deposits, a characteristic of osteogenic cells, in differentiated SK cells, MSC-ad and MSC-BM (Figure 2). ALP, an osteogenic marker, was also expressed by these three cell types under osteogenic differentiation medium for 2 weeks, as determined by NBT/BICP staining (Figure 2). Expression of four adipogenic genes, C/EBP α, C/EBP β, PPAR-γ, and fatty acid binding protein 4 (FABP4), were up-regulated in these three cell types under adipogenic differentiation medium (Figure 3A), and two osteogenic genes, response gene to complement 32 (RGC32) and collagen 1A1, were highly expressed under osteogenic differentiation condition (Figure 3B). On the other hand, the expression of three major MSC markers, CD73, CD90, and CD105, was decreased in all three cell types under both differentiation conditions (Figure 3A and B).

### Tumourigenicity and metastasis of SK cells

**Figure 4:**
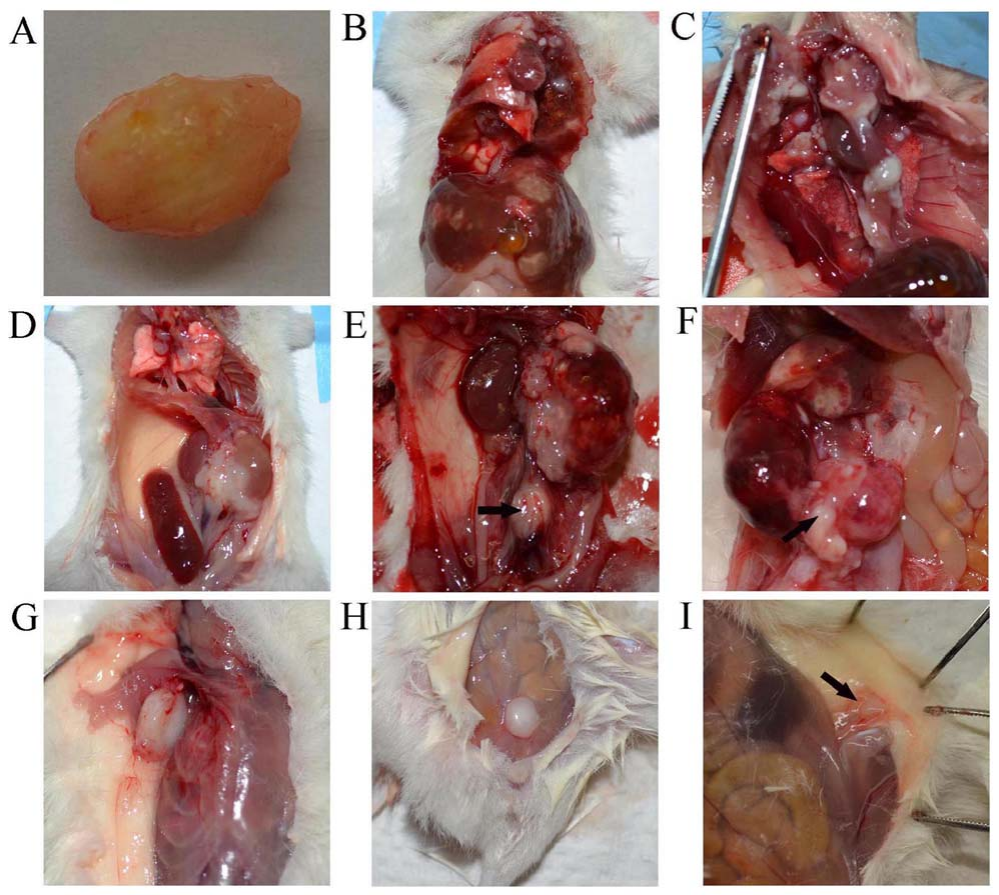
Histological features of primary tumors and metastatic tumors. A, the primary tumors (human xenografts) were formed by the subcutaneous injection of SK cells, and these tumors had a sarcomatous appearance (white color) lacking angiogenesis. B-I, metastatic tumors were found in different organs, such as liver (B), lung (C), spleen, kidney and ureter (D), kidney and ureter (arrow) (E), kidney, liver, and pancreas (arrow) (F), joint at chest and front leg (G), outer peritoneum (H), under skin (arrow) (I) (non-injection site).

**Figure 5:**
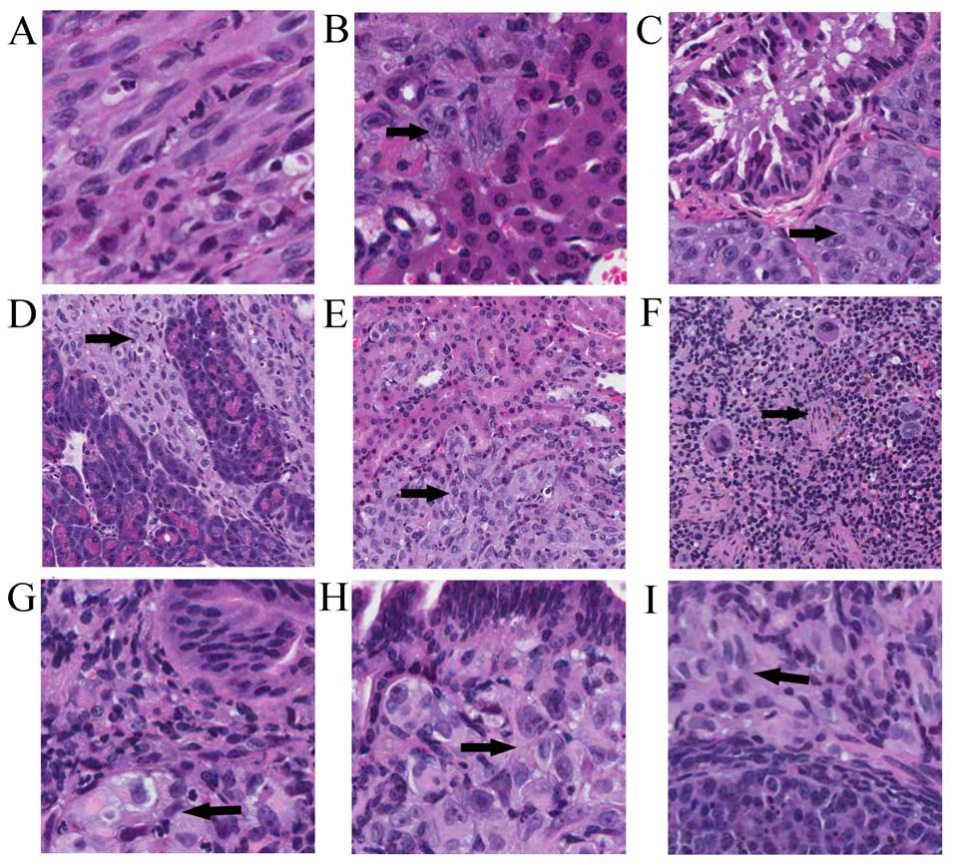
Pathological and histological features of primary tumors and metastatic tumors. A-I, the primary tumors (human xenografts) were formed by the subcutaneous injection of SK cells, and were metastasized to different organs. H and E staining reveals that the histological and pathological features of primary subcutaneous tumor cells (A), metastatic tumor cells (arrow) in liver (B), lung (C), pancreas (D), kidney (E), spleen (F), colon (G), uterus (H), and ovary (I). The tumors consisted of spindle-like shaped cells with ovoid or elongated nuclei containing 2 or more nucleoli per nucleus (A–I). Magnifications: 200 x

Highly immunodeficient mice, NOD/SCID/IL2rg mice were used to evaluate the tumorigenic and metastatic potential of SK cells by us. What surprised us was that as few as 10 SK cells caused tumors in over 96% of injected mice, and even a single cell generated tumors in 10 of 28 injected mice (Table 1). The time period of the tumor formation by injection with a single cell did not show significant difference when compared to higher cell numbers, indicating that SK cells are extremely tumorigenic in NOD/SCID/IL2rg mice. The tumors invaded into muscles during formation and growth; thus, even though the actual tumor size was rather big, the tumor appeared to be smaller. Only after the skin was excised and the tumors were exposed, then the real size of the tumors could be observed (Figure S2A–D). Even sometimes mice were indicated to have the tumors by the fact that mouse back legs could not stand and lost partial function. The mice significantly lost weight within 2 months after injection of SK cells, the average weight of the mice was 18.3±0.73 g in the range of 22.22 g to 15.58 g when they were sacrificed. The interesting finding was that the tumors were widely metastatic, even in the mice injected with a single cell, although the extent of the metastases depended on the number of injected cells within the period of experiments. A few organs were involved in the mice with injection of a single cell, multiple organs had metastases in the mice with injection of ten cells, and widely metastatic lesions occurred in the mice with injection of 100 or more cells (Table 1, Figure 4, Figure S2). These tumors had a sarcomatous appearance (white color) lacking significant angiogenesis (Figure 4, Figure S2D and E). Multiple organs were metastated in all mice, and metastatic tumors were found in livers and lungs in all treated mice (Figure 4, Figure S2F–I). The third frequent metastated organs were kidney (Figure 4D and E) and up-ureters (Figure 4E, arrow), then another organs with metastatic tumors were spleen (Figure 4D), pancreases (Figure 4F, arrow), joint at chest and front leg (Figure 4G), outer peritoneum (abdomen) (Figure 4H), and skin (non-injection site) (Figure 4I, arrow). Metastatic tumors were also found in colon, uterus and ovaries. H and E staining reveals that the histological and pathological features of primary subcutaneous tumor cells (Figure 5A), metastatic tumor cells (arrow) in liver (Figure 5B), lung (Figure 5C), pancreas (Figure 5D), kidney (Figure 5E), spleen (Figure 5F), colon (Figure 5G), uterus (Figure 5H), and ovary (Figure 5I), and the tumors consisted of spindle-like shaped cells with ovoid or elongated nuclei containing 2 or more nucleoli per nucleus (A–I), similar to those formed in nude mice [17]. The details of the histological and pathological features of these primary and metastatic tumor cells were provided in Figure S3–S5.

**Table 1:** Tumourigenicity and metastasis of SK cells and its xenograft cells.

Injected cell types	No. of injected cells	No. of mice with tumors	Days of tumor formation	Metastasis
SK cells	10,000	6/6	33–42	Yes
SK cells	5,000	6/6	37–45	Yes
SK cells	1,000	6/6	37–45	Yes
SK cells	500	6/6	40–46	Yes
SK cells	100	6/6	40–46	Yes
SK cells	10	27/28	40–53	Yes
SK cells	1	10/28	49–64	Yes
Isolates-skin	100	6/6	35–42	Yes
Isolates-liver	100	6/6	35–42	Yes
Isolates-lung	100	6/6	35–40	Yes
Isolates-skin	10	6/6	39–50	Yes
Isolates-liver	10	6/6	41–53	Yes
Isolates-lung	10	6/6	43–54	Yes

The tumourigenicity was evaluated by the injection of SK cells into NOD/SCID/IL2rg mice, and serial transplantation was also performed by the injection of the tumor xenograft cells isolated from primary and metastatic tumors. The tumors were formed, and metastasis was widely found in the mice.

### Re-cultured cells of primary and metastatic tumors and serial transplantation

**Figure 6:**
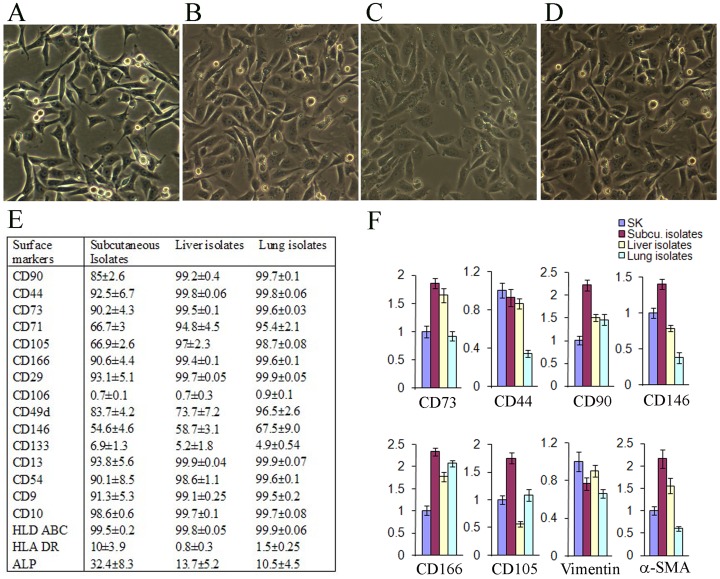
Characterization of isolates from primary tumors and metastatic tumors. A–D, parental SK cells (A), re-culture of subcutaneous primary tumors (B), re-culture of metastatic tumors in liver (C) and lung (D). E, classical mesenchymal stem cell (MSC) markers were detected and measured in three isolates employing flow cytometry. F, expression of MSC markers, vimentin (a mesodermal origin marker), and alpha smooth muscle actin (α-SMA) was measured by quantitative PCR in three isolates. Abbreviations: HLA ABC, major histocompatibility complex (MHC) class I antigen; HLA DR, MHC class II antigen; ALP, Alkaline phosphatase. Magnifications: 100X

The primary tumors produced by the subcutaneous injection, and metastatic tumors in lung and liver were collected and the cells from these tumors were isolated and re-cultured. The morphology of these cells was the same as those of parental SK cells (Figure 6A–D), and flow cytomery results showed that the percentage of classic MSC surface markers in these isolates are very similar to those in parental SK cells (Figure 6E, Figure 6S), but HLA DR were slightly decreased in isolates of livers and lungs. Expression of major MSC markers were not significantly changed (Figure 6F). These isolates from SK xenografts (primary and metastatic tumors) were subcutaneously injected into mice, and subcutaneous tumor formation and metastases occurred as similar to the parental SK cells (Table 1).

### In vitro invasiveness

**Figure 7:**
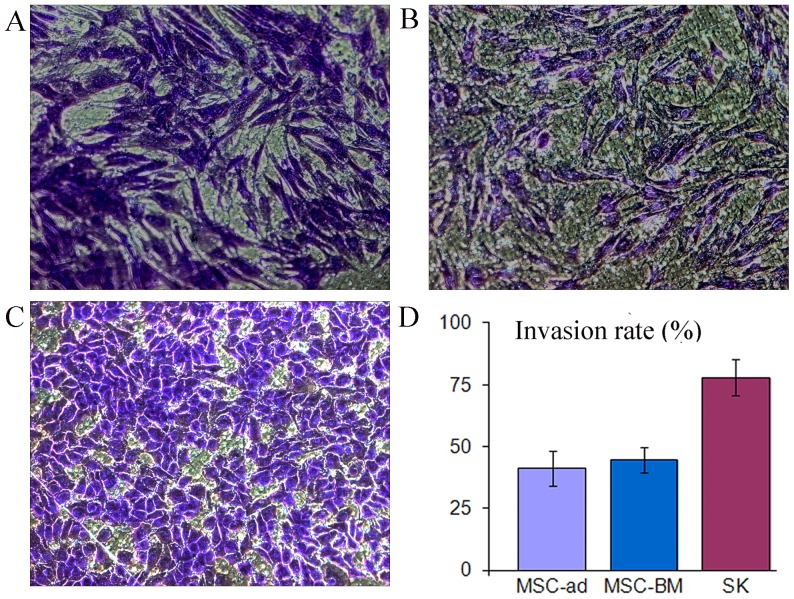
In vitro invasion assay A–C, the invasion of stained adipose-derived msenchymal stem cells (A), bone marrow-derived mesenchymal stem cells (B), and SK cells (C), were evaluated from transwells at 24 hours after culturing under low serum. D, based on the standard curve, the number of invading cells was quantitated. Magnifications: 100× (A–D).

An in vitro invasion assay showed that SK cells had greater migration and invasion capacity when compared to MSC-ad and MSC-BM, a well described invasive cell type. Large number of SK cells passed the basement membrane 24 hours under low FBS conditions, as determined by microscopy after staining (Figure 7A–C). A quantitative assay showed that over 75% of SK cells migrated and went through the membrane within 24 hours, whereas 40% of MSC-ad and MSC-BM passed the membrane during the same period (Figure 7D), this great migration and invasion capacity might explain why mice had widely metastatic lesions after the in vivo injection of the SK cells.

## Discussion

We have determined that SK Hep-1 cells expressed almost all MSC markers, such as CD9, CD10, CD13, CD29, CD49d, CD54, CD71, CD73, CD146, CD166, CD90, CD44, CD105, HLA-ABC, and alkaline phosphatase (ALP) [18], [19]. Although MSC and endothelial cells share some surface markers, endothelial cells cannot be differentiated into adiopogenic and osteogenic lineages, and our results demonstrated that SK cells could be differentiated into these two cell lineages, which is the cardinal property of MSC. SK cells expressed almost all MSC genes, but did not express albumin or alpha fetoprotein (liver lineage), myogenic factor 5 (MYF5), myogenin (MYOG) and desmin (myoblast lineage), glial fibrillary acidic protein (GFAP, hepatic stellate cell lineage), CK14, and CK17 (myoepithelia cell lineage), or CD34, and CD45 (hematopoietic cell lineage). von Willebrand factor (vWF) was slightly expressed in SK cells (ct values were between 32–35 as determined by quantitative PCR); however, other endothelial cell markers, CD34, CD31, CD144, E-selectin, and CD106/ICAM-1, were not detected in SK cells. SK cells have been thought to be endothelial in origin with the major evidence being the presence of Weibel-Palade bodies and tubular formation by the cells [10]. If SK cells were truly of endothelial origin, it is possible to speculate that oncogenically transformed endothelial progenitors might be transdifferentiated into mesenchymal cells after the oncogenic hit, similar to this occurrence during normal embryonic development [13]. However, a more likely explanation of the data suggests the possibility that SK cells might originate from pericyte/perivascular cells. Pericytes and endothelial progenitor cells (EPC) share many properties; for example, they come from mesenchymal origin, share many surface markers, form tubular structures in vitro, and this tubular formation can be inhibited by the same inhibitors [20]. Importantly, pericytes can give rise to MSC in vivo and in vitro [21]. Whether SK cells originated from endothelia cells, or pericytes, or from another cell type of mesenchymal origin is under further investigation.

SK cells were isolated from a patient with liver adenocarcinoma [11], and designated as a hepatoma cell line. In our study, the tumors could be formed within two months after subcutaneous injection of as few as one SK cell in NOD/SCID/IL2rg mice. Normally a single cancer cell does not have the capacity to form tumors, even in highly immnuodeficient mice, only cancer stem cells have this capacity. Importantly, the time period of tumor formation by a single cell did not differ significantly when compared to tumors formed by the injection with 10,000 cells, thus suggesting that SK cells are extremely tumorigenic. Only 10 of 28 mice injected with one cell formed tumors, and the reason may well be because the cell was lost during transplantation and did not actually get into the mouse, or the cell was dead during the preparation and transplantation. Wide-spread metastases were found in the mice, even with a single cell subcutaneously injected, demonstrating aggressive migration and invasion properties which are often associated with cells of mesenchymal origin. This property was confirmed by in vitro invasion assay which showed that over 75% of SK cells could invade base membrane of transwells within 24 h. The cells isolated from primary subcutaneous tumors, or from the metastatic tumors of livers and lungs, did not change their gene expression pattern and MSC phenotype, tumorigenicity and metastatic capacity. Therefore, the entire SK cells population appears to function as a cancer stem cell line with highly tumorigenic and metastatic capacities, and this property can be maintained in in vitro culture and in vivo by serial transplantations.

Normally MSC can maintain multipoptency and their phenotype for a short period at low passage after isolation, and they will lose this property and differentiate or transdifferentiate into other cell types after long-term culture at higher passages in vitro. SK cells appear to function as oncogenically transformed MSC with enhanced self-renewal and proliferation, because they can be unlimitedly expanded and maintained in culture without losing multipotency and changing its phenotype. Although the concept of cancer stem cells is still controversial, recent reports indicate that CSCs do exist in tumors employing mouse models [22]–[26]. However, the origin and formation of cancer stem cells remains elusive, and some reports indicate that cancer stem cells may derive from normal stem cells by acquiring an oncogenic hit [27]–[29]. For example, a recent paper has suggested that liver cancer stem cells are derived from the enhanced self-renewal of facultative liver stem cells [30]. Although cancer stem cells/tumor-initiating cells have been isolated and expanded in culture [26], [31], CSCs have not been shown previously to suscessfully be expanded and be maintained in vitro for long term culture. Thus, we believe that this is the first report to show that SK cells appear to exhibit the characteristics of transformed normal MSC by acquiring enhanced self-renewal capacity after an oncogenic hit, and furthermore, they appear to have transformed into a cancer stem cells line which can be expanded and maintained in vitro and in vivo for a prolonged period without losing MSC phenotype, tumouriogenicity, and metastatic capacity.

The primary tumors or the xenografts produced by cancer stem cells or tumor-initiating cells represent heterogeneous population with different cell types, only a small number of cells remain cancer stem cell property. However, the subcutaneous tumors or metastatic tumors produced by a single SK cells or 10,000 SK cells exhibited a relative homogenous population with MSC phenotype which is the same with parental SK cells. Parental SK cells with MSC characteristics did not differentiate into other cell lineages such as adipose, osteoblast, or chondrocytes during the tumor formation or metastasis after injection into mice. The formation of the subcutaneous tumors and metastatic tumors is just proliferation or self-renewal of SK cells in vivo as they do in vitro. This phenomenon or mechanism is apparently different from those of epithelial cell- and endothelial cell-based tumor/cancer formation and metastasis.

In summary, parental SK cells and their xenografts exhibit same oncogenic MSC characteristics with highly metastatic capacity, even a single SK cell has such tumorigenicity and metastatic capacity, moreover, parental SK cells and their xenografts present a relative homogeneity with the same phenotype. SK cells' mesenchymal origin and mesenchymal phenotype demonstrated that mesenchymal cell type contributed to the liver carcinoma of the patient which SK cells was derived from. Therefore, it represents a novel mechanism of tumor initiation, development and metastasis by cancer stem cells of non-epithelial and endothelia origins.

## Supporting Information

Figure S1 **Characterization of surface markers of SK Hep-1 cells and mesenchymal stem cells.** Flow cytometry was employed to detect and measure classical mesenchymal stem cell (MSC) markers in three cell types, SK Hep-1 cells, adipose-derived MSC (MSC-ad), and bone marrow-derived MSC (MSC-BM).
(JPG)

Figure S2 **The tumor growth and metastasis characteristics.** A, the tumor appearance before open of the skin when the mouse was sacrificed, and the tumor apparently looked small. B, the tumor appearance after open of the skin before the isolation, the tumors grew invasively. C, the tumors were isolated from the mice, and embedded holes were left after the tumors were removed. D, this tumor sizes:>0.5 cm (height) ×1 cm (width) ×1.5 cm (length). E, the average tumor sizes were ≥0.5–1.0 cm (height) ×1.0–1.5 cm (width) ×1.5–2.0 cm (length) after isolation. F, Metastatic tumors were found in multiple organs in single mouse, for example, metastatic tumors were found in lung and liver in one mouse (left), and metastatic tumors were also found in kidney and up-ureter in the same mouse. Arrow indicates normal ureter (right). G, lungs, liver, and kidneys with metastatic tumors were isolated from this single mouse shown in F. H and I: metastatic tumors were found in livers (H) and lungs (I) of all treated mice.
(JPG)

Figure S3 **Histological features of primary tumors and metastatic tumors.** A–C, H and E staining reveals that the histological and pathological features of primary subcutaneous tumor cells (A), metastatic tumor cells (arrow) in liver (B), and lung (C). Magnifications: 40 x.
(JPG)

Figure S4 **Histological features of metastatic tumors.** A–C, H and E staining reveals that the histological and pathological features of metastatic tumor cells (arrow) in pancreas (A), kidney (B), and spleen (C). Magnifications: 40 x.
(JPG)

Figure S5 **Histological features of metastatic tumors.** A–C, H and E staining reveals that the histological and pathological features of metastatic tumor cells (arrow) in colon (A), uterus (B), and ovary (C). Magnifications: 40 x.
(JPG)

Figure S6 **Characterization of surface markers of primary tumor cells and metastatic tumor cells.** Flow cytometry was employed to detect and measure classical mesenchymal stem cell (MSC) markers in three isolates from the primary tumors (Skin-isolate) produced by the subcutaneous injection, and metastatic tumors in liver (Liver-isolate) and lung (Lung-isolate).
(JPG)

Table S1 **List of antibodies used.** Abbreviations: ALP, Alkaline phosphatase; PE, phycoerthrin; HLA ABC, human major histocompatibility complex (MHC) class I, human leukocyte antigens, A, B, C; HLA DR, MHC class II cell surface receptor; FC, flow cytometry.
(DOC)

Table S2 **Information of primers and probes used for qPCR.** Abbreviations: GAPDH, glyceraldehyde-3-phosphate dehydrogenase; AFP, alpha fetoprotein; α-SMA, alpha smooth muscle actin; FABP4, fatty acid binding protein 4; RGC32, response gene to complement 32 protein; COL1A1, Collagen, type I, alpha 1; MYF5, Myogenic factor 5; MYOG, Myogenin or myogenic factor 4; GFAP, Glial fibrillary acidic protein; E-SELE, E-selectin; vWF, Von Willebrand factor; C/EBP, CCAAT-enhancer-binding protein; PPAR, Peroxisome proliferator-activated receptor.
(DOC)
