# Supplementary figures and images for: Hepatoma SK Hep-1 Cells Exhibit Characteristics of Oncogenic Mesenchymal Stem Cells with Highly Metastatic Capacity
Source: PLoS One. 2014 Oct 22;9(10):e110744. doi: 10.1371/journal.pone.0110744 (PMC4206444; doi:10.1371/journal.pone.0110744)

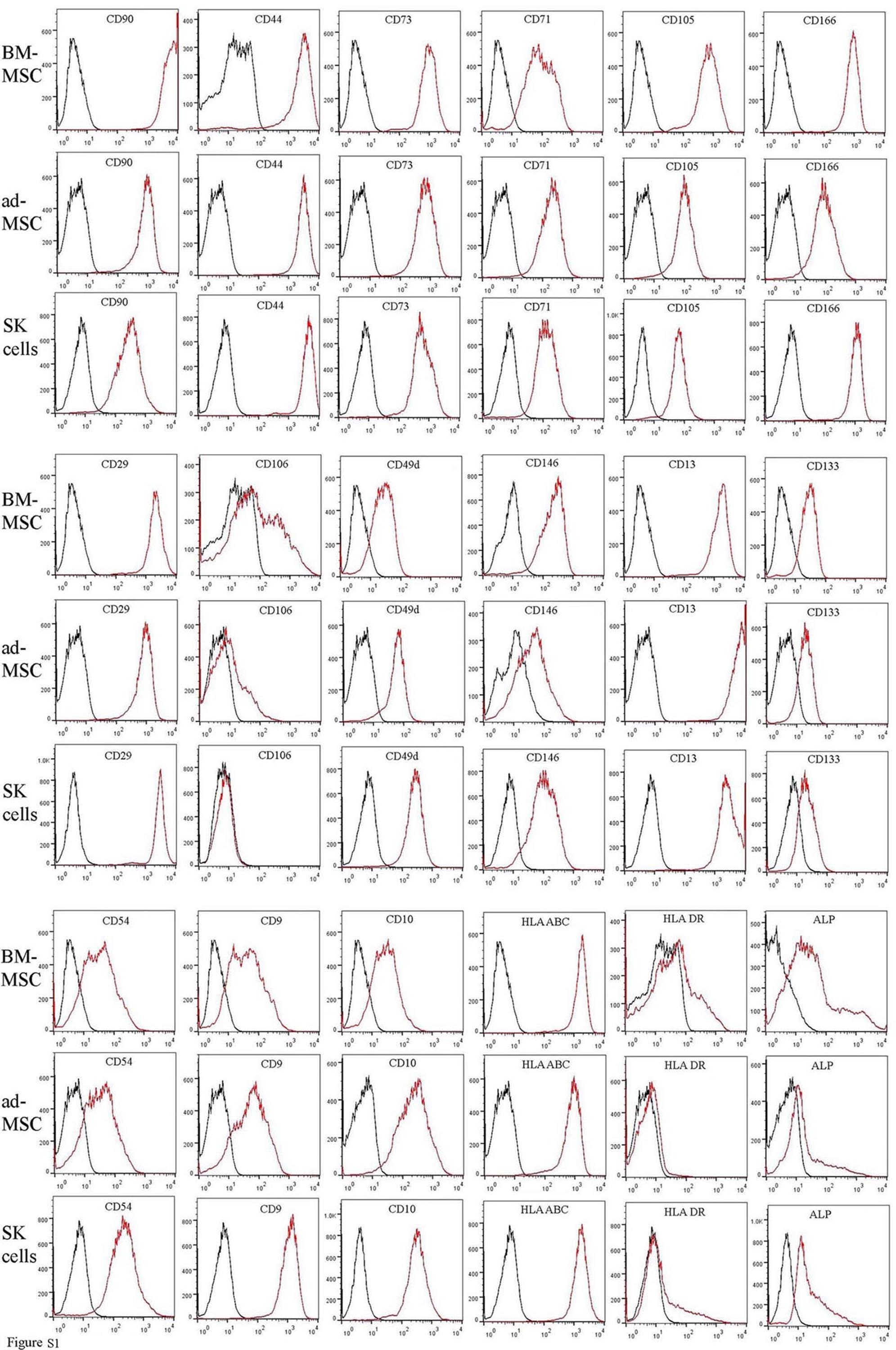

Supplement: Figure S1 — Characterization of surface markers of SK Hep-1 cells and mesenchymal stem cells. Flow cytometry was employed to detect and measure classical mesenchymal stem cell (MSC) markers in three cell types, SK Hep-1 cells, adipose-derived MSC (MSC-ad), and bone marrow-derived MSC (MSC-BM). (JPG) [file pone.0110744.s001.jpg]

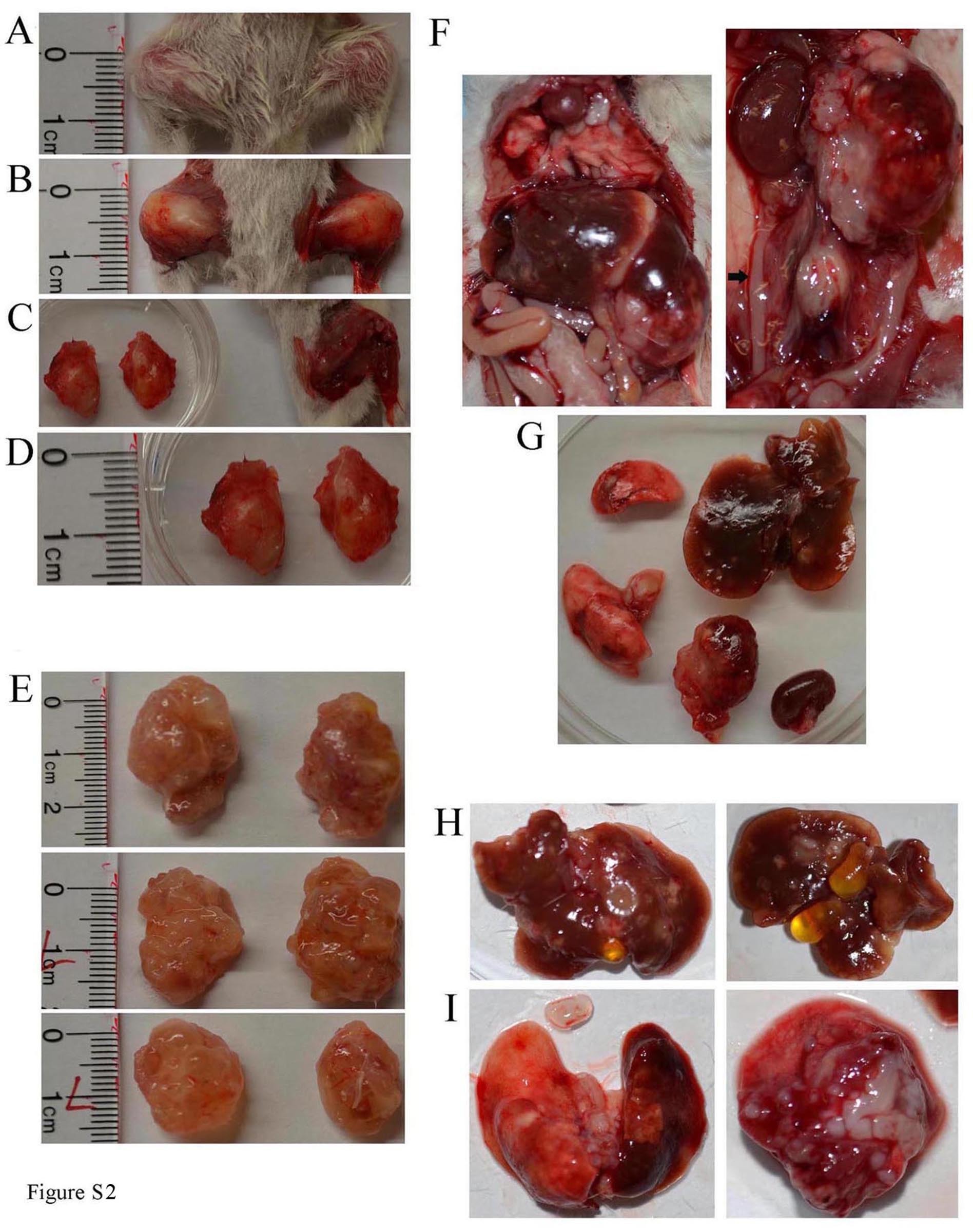

Supplement: Figure S2 — The tumor growth and metastasis characteristics. A, the tumor appearance before open of the skin when the mouse was sacrificed, and the tumor apparently looked small. B, the tumor appearance after open of the skin before the isolation, the tumors grew invasively. C, the tumors were isolated from the mice, and embedded holes were left after the tumors were removed. D, this tumor sizes:>0.5 cm (height) ×1 cm (width) ×1.5 cm (length). E, the average tumor sizes were ≥0.5–1.0 cm (height) ×1.0–1.5 cm (width) ×1.5–2.0 cm (length) after isolation. F, Metastatic tumors were found in multiple organs in single mouse, for example, metastatic tumors were found in lung and liver in one mouse (left), and metastatic tumors were also found in kidney and up-ureter in the same mouse. Arrow indicates normal ureter (right). G, lungs, liver, and kidneys with metastatic tumors were isolated from this single mouse shown in F. H and I: metastatic tumors were found in livers (H) and lungs (I) of all treated mice. (JPG) [file pone.0110744.s002.jpg]

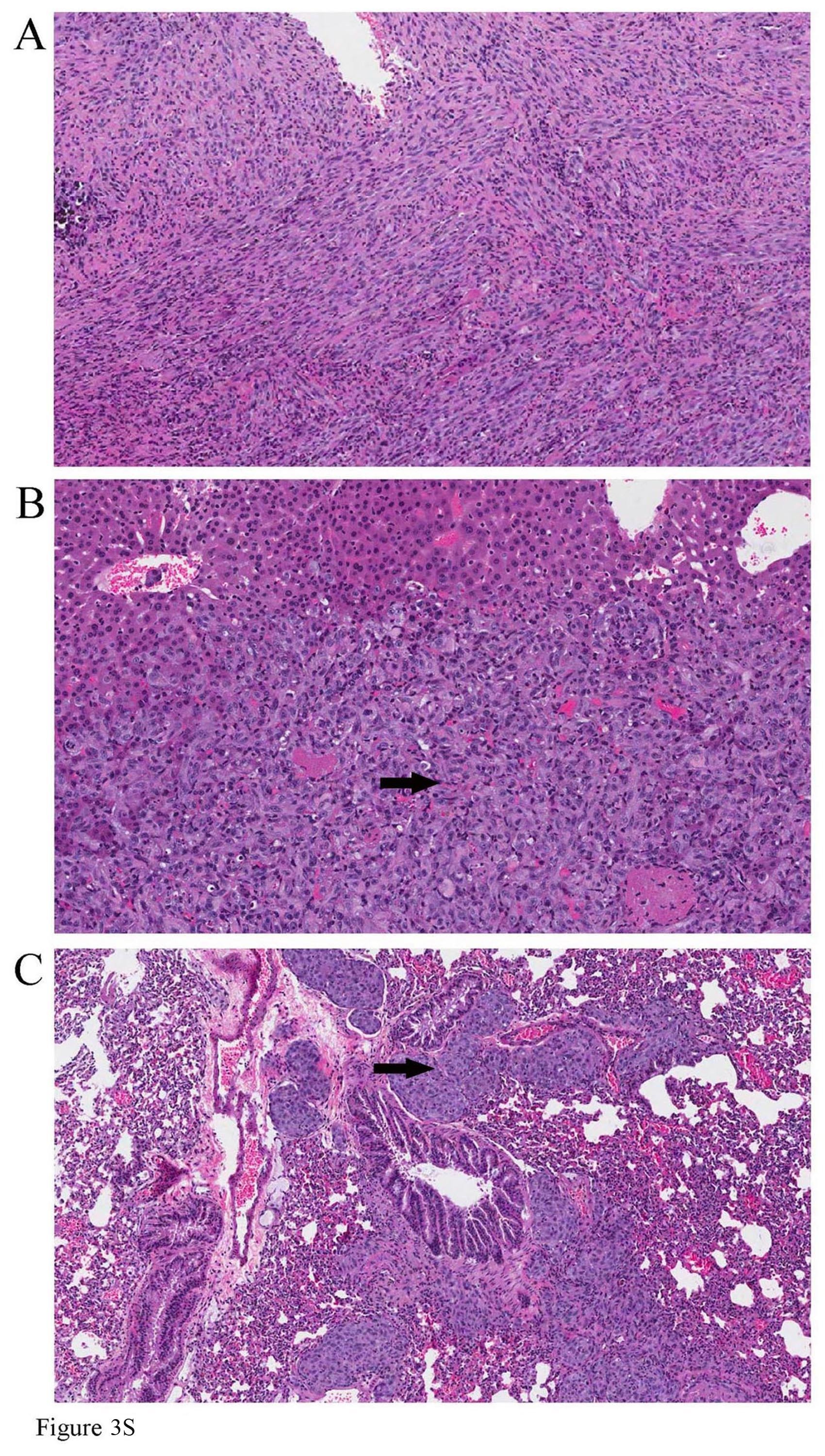

Supplement: Figure S3 — Histological features of primary tumors and metastatic tumors. A–C, H and E staining reveals that the histological and pathological features of primary subcutaneous tumor cells (A), metastatic tumor cells (arrow) in liver (B), and lung (C). Magnifications: 40 x. (JPG) [file pone.0110744.s003.jpg]

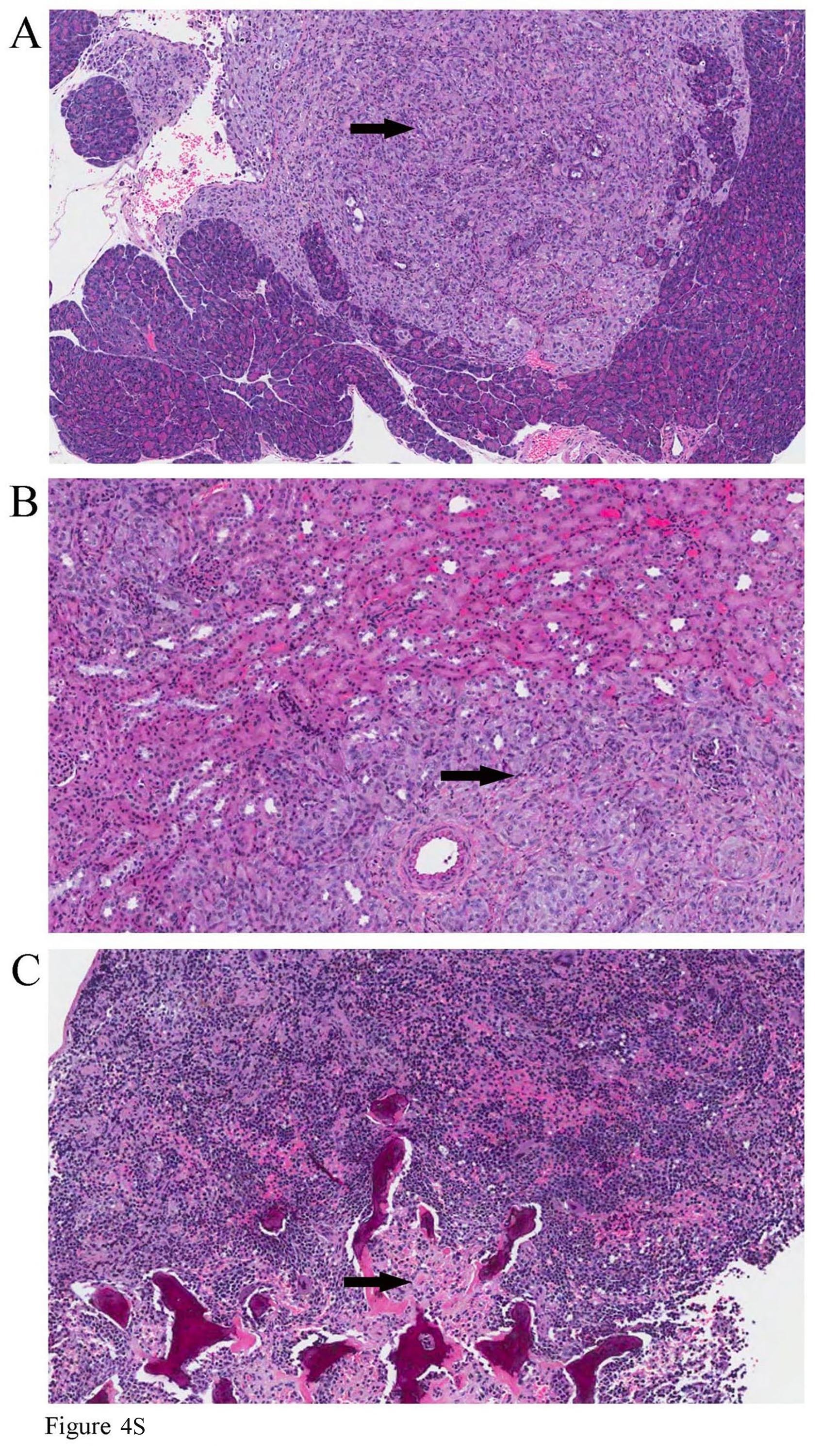

Supplement: Figure S4 — Histological features of metastatic tumors. A–C, H and E staining reveals that the histological and pathological features of metastatic tumor cells (arrow) in pancreas (A), kidney (B), and spleen (C). Magnifications: 40 x. (JPG) [file pone.0110744.s004.jpg]

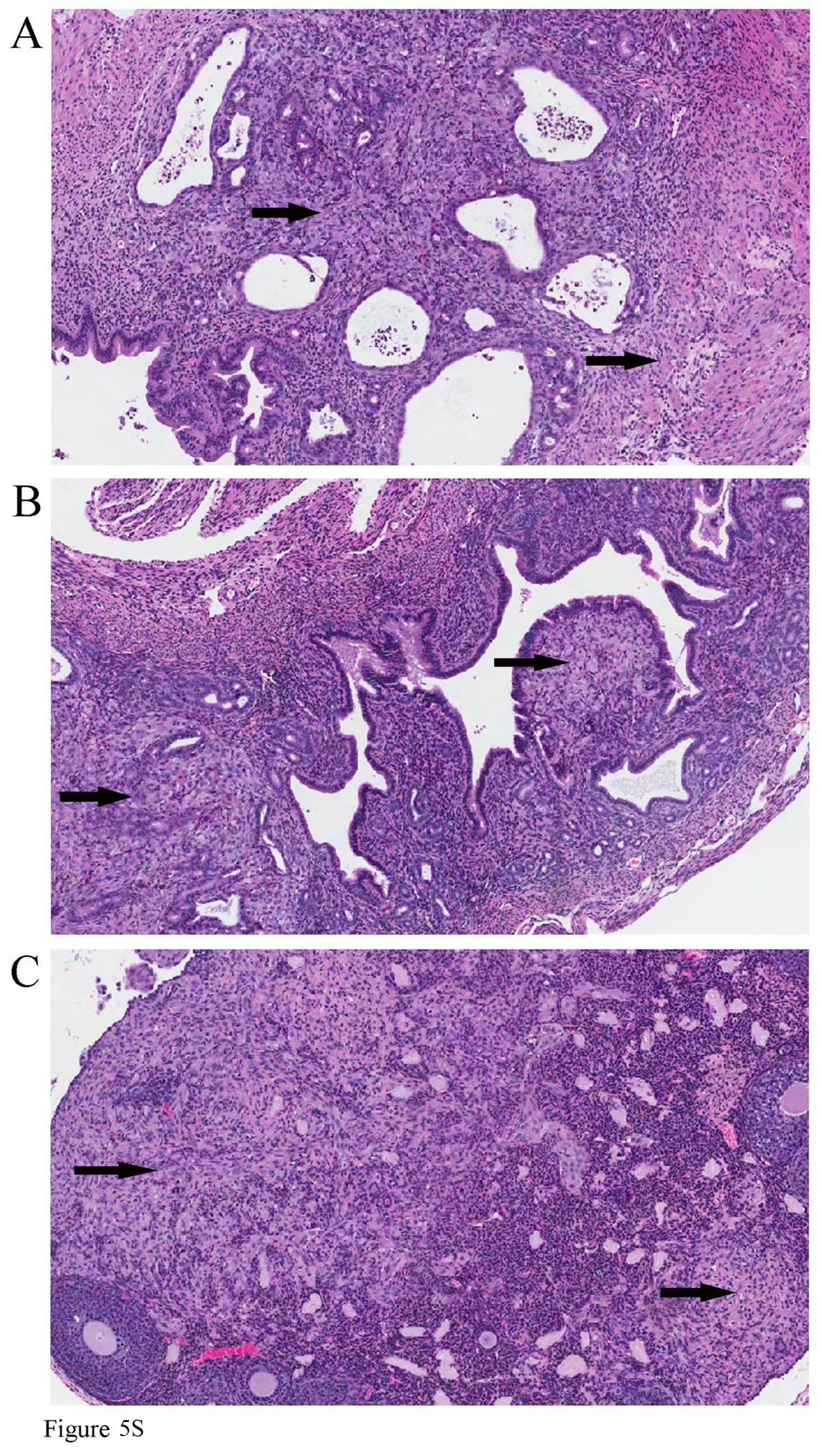

Supplement: Figure S5 — Histological features of metastatic tumors. A–C, H and E staining reveals that the histological and pathological features of metastatic tumor cells (arrow) in colon (A), uterus (B), and ovary (C). Magnifications: 40 x. (JPG) [file pone.0110744.s005.jpg]

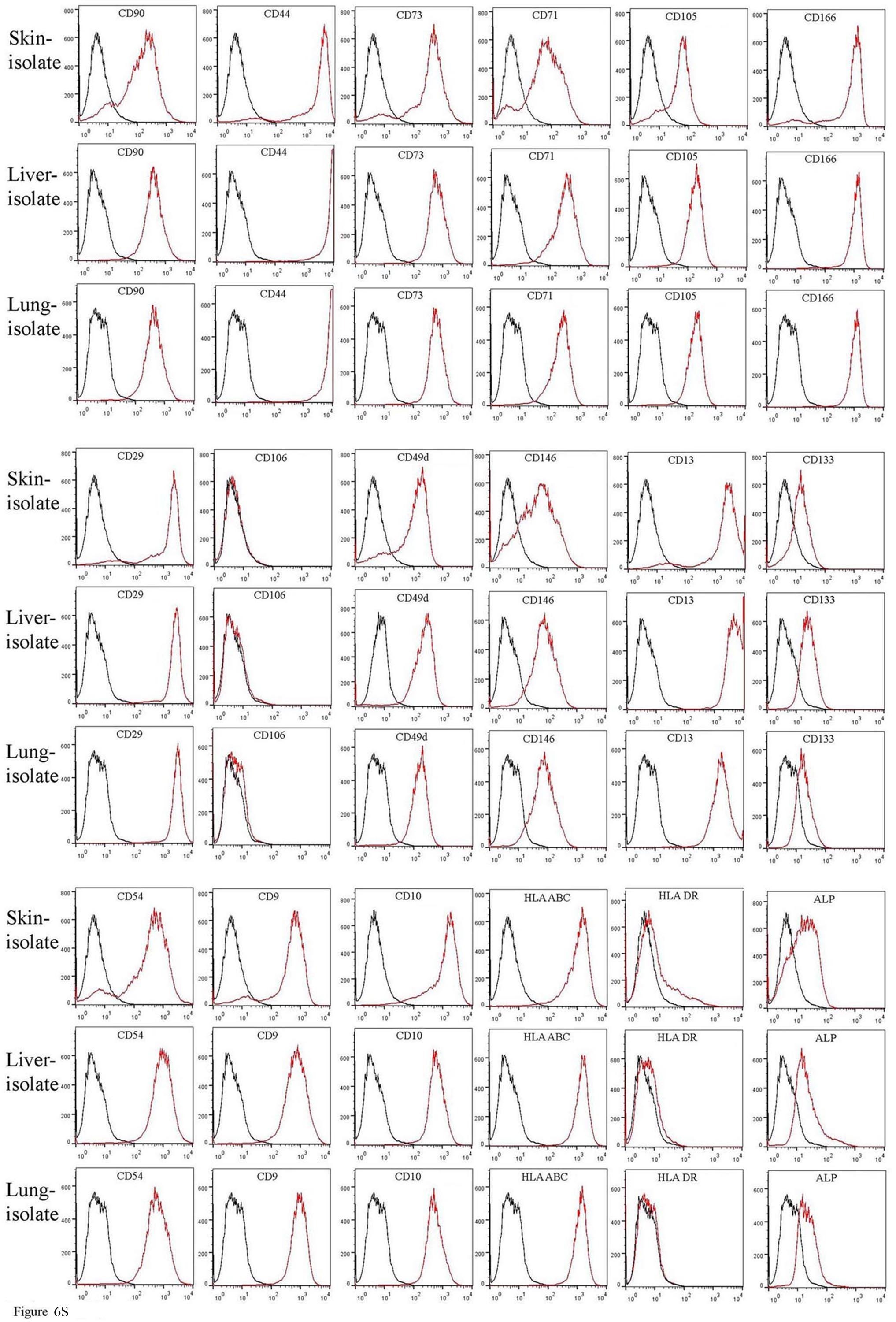

Supplement: Figure S6 — Characterization of surface markers of primary tumor cells and metastatic tumor cells. Flow cytometry was employed to detect and measure classical mesenchymal stem cell (MSC) markers in three isolates from the primary tumors (Skin-isolate) produced by the subcutaneous injection, and metastatic tumors in liver (Liver-isolate) and lung (Lung-isolate). (JPG) [file pone.0110744.s006.jpg]
