# Supplementary material for: Hepatoma SK Hep-1 Cells Exhibit Characteristics of Oncogenic Mesenchymal Stem Cells with Highly Metastatic Capacity
Source: PLoS One. 2014 Oct 22;9(10):e110744. doi: 10.1371/journal.pone.0110744 (PMC4206444; doi:10.1371/journal.pone.0110744)
Supplement: Table S1 — List of antibodies used. Abbreviations: ALP, Alkaline phosphatase; PE, phycoerthrin; HLA ABC, human major histocompatibility complex (MHC) class I, human leukocyte antigens, A, B, C; HLA DR, MHC class II cell surface receptor; FC, flow cytometry. (DOC) [file pone.0110744.s007.doc]

**Supplemental Table S1. List of antibodies used**

| Antigen | Type | Provider | Application |
| --- | --- | --- | --- |
| CD73 | PE conjugated mouse monoclonal | BD Science | FC |
| CD90 | PE conjugated mouse monoclonal | eBioScience | FC |
| CD44 | PE conjugated mouse monoclonal | BD Science | FC |
| CD71 | PE conjugated mouse monoclonal | BD Science | FC |
| CD105 | PE conjugated mouse monoclonal | BD Science | FC |
| CD166 | PE conjugated mouse monoclonal | BD Science | FC |
| CD29 | PE conjugated mouse monoclonal | BD Science | FC |
| CD106 | PE conjugated mouse monoclonal | BD Science | FC |
| CD49d | PE conjugated mouse monoclonal | eBioScience | FC |
| CD146 | PE conjugated mouse monoclonal | BD Science | FC |
| CD9 | PE conjugated mouse monoclonal | eBioScience | FC |
| CD10 | PE conjugated mouse monoclonal | BD Science | FC |
| ALP | PE conjugated mouse monoclonal | BD Science | FC |
| HLA ABC | PE conjugated mouse monoclonal | eBioScience | FC |
| HLA DR | PE conjugated mouse monoclonal | eBioScience | FC |
| CD45 | PE conjugated mouse monoclonal | eBioScience | FC |
| CD34 | PE conjugated mouse monoclonal | BD Science | FC |
| CD90 | PE conjugated mouse monoclonal | eBioScience | FC |
| CD133 | PE conjugated mouse monoclonal | Miltenyl Biotech | FC |
| CD31 | PE conjugated mouse monoclonal | BD Science | FC |
| CD54 | PE conjugated mouse monoclonal | BD Science | FC |
| CD13 | PE conjugated mouse monoclonal | BD Science | FC |
|  | Purified goat IgG | Bethyl | Isotype control |
|  | Purified rabbit IgG | Bethyl | Isotype control |
|  | Purified mouse IgG | Bethyl | Isotype control |

Abbreviations: ALP, Alkaline phosphatase; PE, phycoerthrin; HLA ABC, human major histocompatibility complex (MHC) class I, [human leukocyte antigens](http://en.wikipedia.org/wiki/Human_leukocyte_antigens), A, B, C; HLA DR, [MHC class II](http://en.wikipedia.org/wiki/MHC_class_II) [cell surface receptor](http://en.wikipedia.org/wiki/Cell_surface_receptor); FC, flow cytometry.
