# Supplementary material for: Hepatoma SK Hep-1 Cells Exhibit Characteristics of Oncogenic Mesenchymal Stem Cells with Highly Metastatic Capacity
Source: PLoS One. 2014 Oct 22;9(10):e110744. doi: 10.1371/journal.pone.0110744 (PMC4206444; doi:10.1371/journal.pone.0110744)
Supplement: Table S2 — Information of primers and probes used for qPCR. Abbreviations: GAPDH, glyceraldehyde-3-phosphate dehydrogenase; AFP, alpha fetoprotein; α-SMA, alpha smooth muscle actin; FABP4, fatty acid binding protein 4; RGC32, response gene to complement 32 protein; COL1A1, Collagen, type I, alpha 1; MYF5, Myogenic factor 5; MYOG, Myogenin or myogenic factor 4; GFAP, Glial fibrillary acidic protein; E-SELE, E-selectin; vWF, Von Willebrand factor; C/EBP, CCAAT-enhancer-binding protein; PPAR, Peroxisome proliferator-activated receptor. (DOC) [file pone.0110744.s008.doc]

**Supplemental Table S2. Information of primers and probes used for qPCR**

| Genes | Information or sequences of primers | Application |
| --- | --- | --- |
| Albumin | Hs00609411_m1 (Applied Biosystems) | TaqMan |
| CD73 | Hs00159686_m1 (Applied Biosystems) | TaqMan |
| GAPDH | Hs99999905_m1 (Applied Biosystems) | TaqMan |
| CK14 | Hs00265033_m1 (Applied Biosystems) | TaqMan |
| CK17 | Hs01588578_m1 (Applied Biosystems) | TaqMan |
| CD34 | Hs00990735_m1 (Applied Biosystems) | TaqMan |
| AFP | Hs00173490_m1 (Applied Biosystems) | TaqMan |
| CD44 | Hs01075861_m1 (Applied Biosystems) | TaqMan |
| CD146 | Hs00920938_g1 (Applied Biosystems) | TaqMan |
| CD31 | Hs00169777_m1 (Applied Biosystems) | TaqMan |
| CD166 | Hs00977639_m1 (Applied Biosystems) | TaqMan |
| CD90 | Hs00264235_s1 (Applied Biosystems) | TaqMan |
| CD105 | Hs00923996_m1 (Applied Biosystems) | TaqMan |
| Vimentin | Hs00185584_m1 (Applied Biosystems) | TaqMan |
| α-SMA | Hs00426835_m1 (Applied Biosystems) | TaqMan |
| FABP4 | Hs01086177_m1 (Applied Biosystems) | TaqMan |
| RGC32 | Hs00204129_m1 (Applied Biosystems) | TaqMan |
| COL1A1 | Hs00164004_m1 (Applied Biosystems) | TaqMan |
| MYF5 | Hs00929416_g1 (Applied Biosystems) | TaqMan |
| MYOG | Hs01072232_m1 (Applied Biosystems) | TaqMan |
| DES | Hs00157258_m1 (Applied Biosystems) | TaqMan |
| GFAP | Hs00909233_m1 (Applied Biosystems) | TaqMan |
| CD144 | Hs00901463_m1 (Applied Biosystems) | TaqMan |
| E-SELE | Hs00174057_m1 (Applied Biosystems) | TaqMan |
| vWF | Hs00169795_m1 (Applied Biosystems) | TaqMan |
| CD166 | Hs00977639_m1 (Applied Biosystems) | TaqMan |
| CD45 | Hs00894732_m1 (Applied Biosystems) | TaqMan |
| GAPDH | F: 5’-GAAGATGGTGATGGGATTTC-3’  R: 5’-GAAGGTGAAGGTCGGAGTC-3’ | SYBR |
| C/EBP α | F: 5’-CTCGAGGCTTGCCAGACCGT-3’  R: 5’-GCGGGCTTGTCGGGATCTCAG-3’ | SYBR |
| C/EBP β | F: 5’-GCCCTCGCAGGTCAAGAGCA-3’  R: 5’-TTGAACAAGTTCCGCAGGGTG-3’ | SYBR |
| PPAR-γ | F: 5’-TTCAGAAATGCCTTGCAGTG-3’  R: 5’-CCAACAGCTTCTCCTTCTCG-3’ | SYBR |

Abbreviations: GAPDH, glyceraldehyde-3-phosphate dehydrogenase; AFP, alpha fetoprotein; α-SMA, alpha smooth muscle actin; FABP4, fatty acid binding protein 4; RGC32, response gene to complement 32 protein; COL1A1, Collagen, type I, alpha 1; MYF5, Myogenic factor 5; MYOG, Myogenin or myogenic factor 4; GFAP, Glial fibrillary acidic protein; E-SELE, [E-selectin](http://en.wikipedia.org/wiki/E-selectin); vWF, Von Willebrand factor; C/EBP, CCAAT-enhancer-binding protein; PPAR, Peroxisome proliferator-activated receptor.
